# A Lytic *Yersina pestis* Bacteriophage Obtained From the Bone Marrow of *Marmota himalayana* in a Plague-Focus Area in China

**DOI:** 10.3389/fcimb.2021.700322

**Published:** 2021-07-08

**Authors:** Junrong Liang, Shuai Qin, Ran Duan, Haoran Zhang, Weiwei Wu, Xu Li, Deming Tang, Guoming Fu, Xinmin Lu, Dongyue Lv, Zhaokai He, Hui Mu, Meng Xiao, Jinchuan Yang, Huaiqi Jing, Xin Wang

**Affiliations:** ^1^ State Key Laboratory of Infectious Disease Prevention and Control, National Institute for Communicable Disease Control and Prevention, Chinese Center for Disease Control and Prevention, Beijing, China; ^2^ Sanitary Inspection Center, Xuzhou Municipal Centre for Disease Control and Prevention, Xuzhou, China; ^3^ School of Light Industry, Beijing Technology and Business University, Beijing, China; ^4^ Sanitary Inspection Center, Subei Mongolian Autonomous County Center for Disease Control and Prevention, Jiuquan, China; ^5^ Sanitary Inspection Center, Akesai Kazakh Autonomous County Center for Disease Control and Prevention, Jiuquan, China

**Keywords:** bacteriophage, Yersinia pestis, Marmota himalayana, natural plague focus, Qinghai-Tibet plateau

## Abstract

A lytic *Yersinia pestis* phage vB_YpP*-*YepMm (also named YepMm for briefly) was first isolated from the bone marrow of a *Marmota himalayana* who died of natural causes on the Qinghai-Tibet plateau in China. Based on its morphologic (isometric hexagonal head and short non-contractile conical tail) and genomic features, we classified it as belonging to the *Podoviridae* family. At the MOI of 10, YepMm reached maximum titers; and the one-step growth curve showed that the incubation period of the phage was about 10 min, the rise phase was about 80 min, and the lysis amount of the phage during the lysis period of 80 min was about 187 PFU/cell. The genome of the bacteriophage YepMm had nucleotide-sequence similarity of 99.99% to that of the *Y. pestis* bacteriophage Yep-phi characterized previously. Analyses of the biological characters showed that YepMm has a short latent period, strong lysis, and a broader lysis spectrum. It could infect *Y. pestis*, highly pathogenic bioserotype 1B/O:8 *Y. enterocolitica*, as well as serotype O:1b *Y. pseudotuberculosis*—the ancestor of *Y. pestis*. It could be further developed as an important biocontrol agent in pathogenic *Yersinia* spp. infection.

## Introduction

Bacteriophages are the most abundant organisms on earth that can interactions with myriad bacterial hosts (Bergh et al., 1989). Lytic bacteriophages have been used as agents for identification and therapeutic of infections in animals and humans (Mukerjee et al., 1963; Gorski et al., 2009; Muniesa et al., 2012; Chhibber et al., 2013; Moojen, 2013; Doub, 2020). Integrity of the bacteriophage tail is essential for the viability of tailed phages, which belong to the *Caudovirales* (Hardy et al., 2020). The tail protein of *Caudovirales* has an important role in the interaction between bacteriophages and host bacteria, which can serve as an adsorption device, a host cell wall-perforating machine, and a genome delivery pathway (Flayhan et al., 2014; Zhang et al., 2018).

In the bacteria of the genus *Yersinia*, bacteriophages have also been used for typing and diagnostics. Bacteriophages ΦYeO3-12 and phiYe-F10 are specific for the *Yersina enterocolitica* serotype O:3 (Kiljunen et al., 2003; Liang et al., 2016); PhiA1122 and Yep-phi are used as a diagnostic agent to confirm the identification of *Yersina pestis*; YpsP-G and YpP-R have been reported to diagnose *Yersina pseudotuberculosis* infection. Many genomes of *Y. pestis* bacteriophages have been fully sequenced, including the *Podoviridae* bacteriophages phiA1122, Yep-phi, Berlin, Yepe2, YpP-R, YpP-G, YpsP-G, Yps-Y, and the *Myoviridae* bacteriophages L-413C, PY100, YpsP-PST, and phiD1 (Garcia et al., 2003; Kiljunen et al., 2011; Rashid et al., 2012; Zhao and Skurnik, 2016).

**Figure 1:**
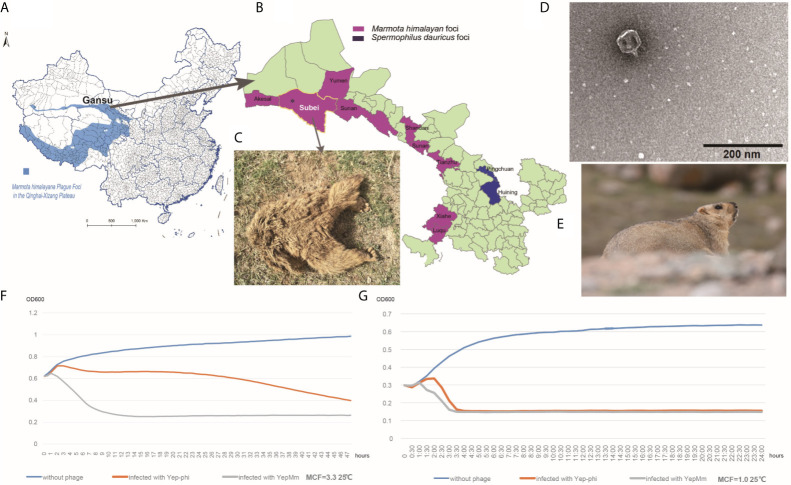
Characteristics of the *Y. pestis* bacteriophage YepMm. **(A)** *Marmota himalayana* in a plague-area focus in Qinghai-Tibet plateau in China. **(B)** *Marmota himalayana* in a plague-area focus in Gansu Province, China. **(C)** The *Marmota himalayana* (who died of natural causes) from which we isolated a lytic *Y. pestis* bacteriophage: YepMm. **(D)** Electron microscopy of YepMm. **(E)** A healthy *Marmota himalayana* in a plague-focus area of the Qinghai-Tibet plateau. **(F)** Growth curves of *Y. pestis* EV76 at 25°C, with MCF = 3.3 in the initial culture. Approximately 3.0 ± 0.2 × 10^7^ PFU of the bacteriophages YepMm and Yep-phi in 30 µl were mixed with 300 µl of the bacterial culture (MCF = 3.3), respectively, and allowed to incubate for 24 h at 25°C. Each group had three duplicates. The OD_600_ value of each group was measured every 30 min. The blue line shows the growth curve of strains without bacteriophage infection. The orange line shows the growth curve of strains infected with the bacteriophage Yep-phi. The gray line shows the growth curve of strains infected with the bacteriophage YepMm. **(G)** Growth curves of *Y. pestis* EV76 at 25°C, with MCF = 1.0 in the initial culture. Approximately 3.0 ± 0.2 × 10^7^ PFU of the bacteriophages YepMm and Yep-phi in 30 µl were mixed with 300 µl of the bacterial culture (MCF = 1.0), and incubated for 24 h at 25°C. Each group had three duplicates. The OD_600_ of each group was measured every 30 min. The blue line shows the growth curve of strains without bacteriophage infection. The orange line shows the growth curve of strains infected with the bacteriophage Yep-phi. The gray line show the growths curve of strains infected with the bacteriophage YepMm.

Qinghai-Tibet plateau is one of the most active natural plague focus in China with *M. himalayana* as the primary host in this area (**Figure 1A**) (**Figure 1E** shows a healthy *Marmota himalayana* in a plague-focus area of the Qinghai-Tibet plateau). The high altitude and harsh climate in the Qinghai-Tibet plateau show that there are few human inhabitants, and the local ecology is relatively stable. Local *M. himalayana* carries a significantly high seropositivity rate of *Y. pestis* F1 antibody, which can be witnessed by continuous outbreaks of plague in animals (*M. himalayana*) and occasionally spreading to humans (Wang et al., 2011; Ge et al., 2015; Wang et al., 2017). With one human case in 2004, two cases in 2007, one case in 2010, and three cases in 2014 (Ge et al., 2015) among the natural-focus area of Qilian Mountain (**Figure 1B**). There is no report about the *Y. pestis* bacteriophage that naturally existed in the host animals of natural plague foci. So we try to isolate *Y. pestis* bacteriophage from different sources in Qinghai-Tibet plateau and investigate the characterization and subsequent employment of the phages.

In the present study, the bacteriophage vB_YpP*-*YepMm obtained from the bone marrow of self-died *Marmota himalayana*. The bacteriophage YepMm could lyse three human pathogenic *Yersinia* species and can be used as a biocontrol agent.

## Materials and Methods

### Bacteriophage Isolation

In the routine prevalence surveillance for *Y. pestis* in China, a *Marmota himalayana* that had died of natural causes (**Figure 1C**) was collected from a plague-focus area in the Qinghai-Tibet plateau in China at an altitude of 3076.85 m (39°52′ N, 95°03′ E). *Yersinia* species-selective Cefsulodin–Irgasan–Novobiocin (CIN) agar (Oxoid, Basingstoke, UK) was used to detect the host strain *Y. pestis*. The *Y. pestis*–specific phage can lyse the host strains to form transparent plaques on it. The phage YepMm and its original host strain (*Y. pestis* dcw-bs-007) were isolated together from the same bone-marrow samples of *M. himalayana*. The lytic bacteriophage (vB_YpP*-*YepMm) was propagated and spotted on CIN agar plates after incubating for 24 h at 25°C. Subsequently, a single-lysis zone of bacteriophage was picked with a sterile truncated tip and amplified in the presence of *Y. pestis* EV76 in *Brucella* medium for 24 h at 37°C. The solution was filtered through a sterile 0.22-μm syringe filter. Afterward, the filtered fluid and EV76 were poured on top of the agar plate to obtain purified bacteriophage.

### Electron Microscopy

Crude bacteriophage lysates (~5×10^10^ PFU/mL) were filter-sterilized using a 0.22-µm membrane (Millipore, Waltham, MA, USA) and then pelleted at 25,000*g* for 1 h at 4°C using a high-speed centrifuge (Beckman Coulter, Palo Alto, CA, USA). The bacteriophage pellet was resuspended in 150 μl of SM-buffer supplemented with CaCl_2_ (5 mM) after washing twice in a neutral solution of ammonium acetate (0.1 M). Bacteriophage particles were deposited onto a carbon-coated Formvar film on copper grids and stained with 20 μl of 2% potassium phosphotungstate (pH 7.2). After dye removal with filter paper, bacteriophage particles were examined under a transmission electron microscope (TECNAI 12; FEI, Hillsboro, OR, USA) at 120 kEv. Images were collected and analyzed using Digital Micrograph™ (Gatan, Pleasanton, CA, USA). Taxonomic assignments were made according to the classification scheme for bacteriophages developed by Ackermann and Berthiaume (Berthiaume and Ackermann, 1977) and the International Committee on the Taxonomy of Viruses.

### Genome Sequencing of Bacteriophage DNA, Assembly, and Bioinformatics Analysis

Bacteriophage DNA was obtained from purified 2.4 × 10^9^ PFU/ml bacteriophage particles as described previously (Shubeita et al., 1987). We tested the quality of the whole genome of bacteriophages with Qubit3.0 (Life Technologies, Carlsbad, CA, USA). A random “shotgun” library was constructed using the NEBNext DNA ultra II protocol. Whole-genome sequencing was carried out using the HiSeq2500 Genome Analyzer (Illumina, San Diego, CA, USA). Generated reads were assembled using the SPAdes algorithm. The average nucleotide identity (ANI) was determined among all pairwise combinations of phage genomes. The assembly sequence was evaluated and corrected with PhageTerm (Hu et al., 2020), putative open reading frame (ORF) was predicted by Prokka 1.1.3. The annotated genome sequence of the bacteriophage YepMm has been deposited into the National Center for Biotechnology Information GenBank database under the accession numbers MW767996 and BankIt 2439990.

### Determination of Host Ranges

The host range of the bacteriophage YepMm was estimated using the classical plaque assay. The infectivity of the membrane-filtered phage lysate (2.4×10^9^ PFU/ml) was tested on the bacterial strains listed in **Table 1**. All experiments with viable *Y. pestis* except EV76 were undertaken in a Biosafety Level-3 laboratory. The formation of lysis zone was determined using a double-layer plaque at 25°C or 37°C after 24 h of incubation.

**Table 1:** Lytic activity of the bacteriophages Yep-phi and YepMm at 37°C and 25°C.

Species	Serotype (Bioserotype for *Y.e*)	Strain	YepMm		Yep-phi	
			37°C	25°C	37°C	25°C
*Y. pestis*	/	Azi30	+	+	+	+
	/	Azi32	+	+	+	+
	/	Azi34	+	+	+	+
	/	Azi36	+	+	+	+
	/	Azi39	+	+	+	+
	/	Azi42	+	+	+	+
	/	EV76	+	+	+	+
*Y. enterocolitica*	1B/0:8	YE92010	+	–	–	–
	1B/0:8	Pa12986	+	+	–	–
	1B/0:8	WA	+	+	–	–
	1B/0:8	52211	+	+	–	–
	1A/O:8	JS2012-xz034	−	−	−	−
	1A/O:8	JS1986-Y40	−	−	−	−
	2/O:9	2 strains	−	−	−	−
	3/O:3	3 strains	−	−	−	−
	1A/O:5,27	3 strains	−	−	−	−
*Y. pseudotuberculosis*	O:14	YP014	+	+	+	+
	O:1a	53512	+	−	−	−
	O:1b	PTB3	+	+	−	−
	O:2a	53517	−	−	−	−
	O:3	YP3	−	−	−	−
	O:3b	YP2B	−	−	−	−
	O:4b	YP4B	−	−	−	−
	O:6	YP6	−	−	−	−
	O:8	YP09	−	−	−	−
	O:10	YO010	−	−	−	−
	O:15	YP15	−	−	−	−
*Escherichia coli*	EPEC	2 strains	−	−	−	−
	EIEC	2 strains	−	−	−	−
	ETEC	2 strains	−	−	−	−
	EAEC	2 strains	−	−	−	−
	EHEC	2 strains	−	−	−	−
*Shigella* species	*Shigella flexneria*	5 strains	−	−	−	−
	*Shigella sonnei*	5 strain	−	−	−	−
*Salmonella* species		10 strains	−	−	−	−

no serotype for Y. pestis.

### Optimal Multiplicity of Infection Determination and One-Step Growth Assays

To estimate MOI, different amounts of phages were serially diluted and incubated with host bacteria EV76 (2 × 10^8^ CFU/ml) (at different MOI of 100, 10, 1, 0.1, 0.01, 0.001) at 37°C for 3 h. After incubation, the phage titer of each MOI phage-host assay group was examined. The highest phage titer group was the optimal MOI. Three parallel experiments were performed for this MOI assay.

The one-step growth assay was carried out as follows: equivalent ratios of overnight cultures of EV76 were mixed with YepMm suspension at an MOI=10. After incubation at 37°C for 15 min, the mixture was centrifuged at 11,000*g* for 30 s. The pellet was then resuspended in 10-ml fresh media. The phage titer was tested with 5-min intervals at the first 30 min and 10-min intervals at the last 90 min by a double-layer agar method.

### Comparison of the Lytic Ability of the Bacteriophages YepMm and Yep-phi

The growth conditions and lytic ability of the bacteriophages YepMm and Yep-phi were tested on host strain *Y. pestis* EV76. EV76 was grown in *Brucella* medium at 27°C to reach McFarland turbidity (MCF) of 3.3 and 1.0, respectively. Each MCF culture solution was divided into three groups (with 300 µl of bacterial culture in each group). Group A was mixed with 30 µl of the bacteriophage YepMm (~3.0 × 10^7^ PFU), group B was mixed with 30 µl of the bacteriophage Yep-phi (~3.2×10^7^ PFU), group C was mixed with 30 µl of phosphate-buffered saline in EV76 culture solution. Each group with three duplicates was allowed to incubate for 48 h, and OD_600_ for each group was measured every 30 min. Experiments were carried out at 25°C and 37°C, respectively. Data are the mean ± SD of three independent experiments.

## Results

### Electron Microscopy and Biological Characteristic

Purified phages YepMm was examined using transmission electron microscopy after negative staining (**Figure 1D**). The virions showed hexagonal outlines with isometric, hexagonal heads and short, noncontractile, conical tails and were classified as members of the *Podoviridae* family.

The optimal multiplicity of infection for phage vB_YpP-YepMm was 10 (**Table S1**), and the one-step growth curve showed that the incubation period of the phage was about 10 min, the rise phase was about 80 min, and the lysis amount of the phage during the lysis period of 80 min was about 187 PFU/Cell (**Figure S1**).

### Sensitivity Test

Three *Y. pseudotuberculosis* strains were sensitive to the bacteriophage YepMm: O:1b and O:14 were sensitive at 25°C and 37°C; O:1a was sensitive at 37°C but not at 25°C. The bacteriophage YepMm could lyse *Y. pestis* and strains of the highly pathogenic *Y. enterocolitica* bioserotype 1B/O:8 at both temperatures (**Table 1**). However, the bacteriophage Yep-phi can only lyse *Y. pestis* and O:14 *Y. pseudotuberculosis.* YepMm can form larger plaques at 25°C than at 37°C (data not shown), indicating (as expected) a temperature-dependent response.

### Genome Sequencing and Bioinformatics Analyses

**Figure 2:**
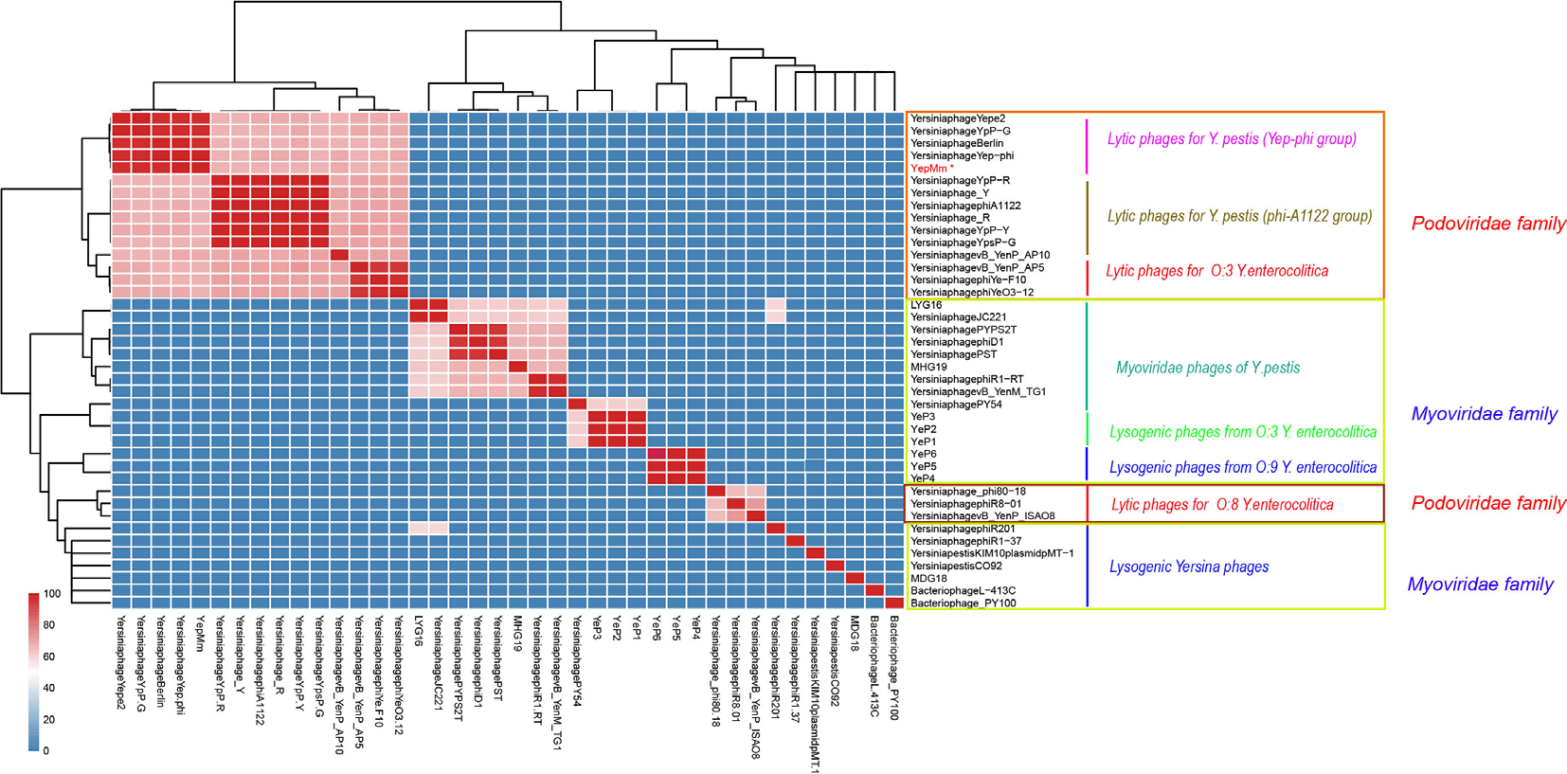
Average nucleotide identity (ANI) matrix of phages. ^∗^Indicates the lytic bacteriophage of *Yersinia pestis* in this study.

**Figure 3:**
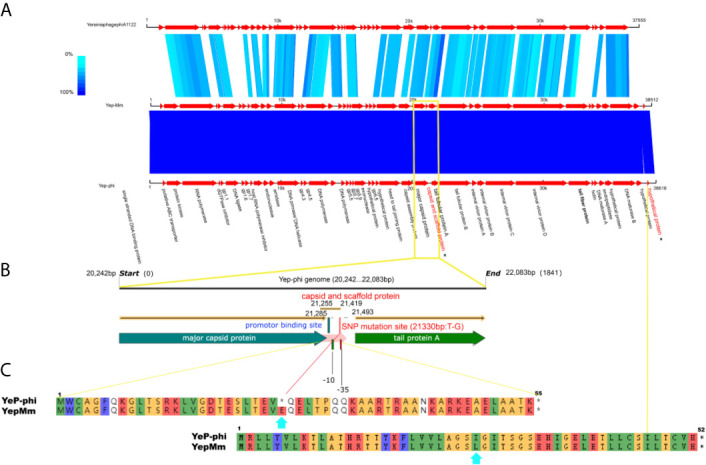
The biological sequence analysis of the bacteriophages YepMm, Yep-phi, and phiA1122. **(A)** Pairwise comparison of the nucleotide sequences of bacteriophages YepMm, Yep-phi, and phiA1122 (the two different ORFs were indicated in asterisks). **(B)** The main difference between the two *Y. pestis* phages of YepMm, Yep-phi. The intergenic region and new ORFs with SNP mutation of YepMm was indicated in yellow rectangle. **(C)** The amino acid similarity alignment of the new ORF-29 of bacteriophages YepMm and Yep-phi.

The complete nucleotide sequence of YepMm is 38,512 bp, with G+C content of 47.1 mol%. It was assembled as a circular molecule and contains no RNA genes. The lysis genes encoding the holin (33,704 to 33,910 bp), endolysin (9,108 to 9,563 bp), and so on existed; no genes associated with lysogenic cycle were founded, such as integrase, lysis repressor. In total, 43 gene products were predicted in the YepMm genome; functions were assigned to 42 of them based on the similarities of the predicted products to known proteins. Genomic comparisons indicated that the genome of some lytic *Y. pestis* phages was highly similar. Bacteriophage YepMm shares 99.99% nucleotide sequence identity with Yep-phi, 97.91% with Berlin, 96.46% with Yepe2, 96.35% with YpP-G, but only 67.48% nucleotide sequence identity with phiA1122 (**Figure 2**). The genome sequences of YepMm and Yep-phi had exactly similar genetic organization, which all contain 222-bp direct repeats at the termini of the mature DNAs and both had head and tail genes in the same relative positions. There are 43 new open reading frame (ORFs) in genome sequence of YepMm and 41 ORFs are 100% identical to Yep- phi, except for the new ORF -29 (phage capsid and scaffold, 21,255 to 21,419 bp) and ORF-43 (**Figure 3A** and **Table S2**). All together, the mutations were primarily for 104 bp deletions in the intergenic and six short nucleotide polymorphisms (SNPs) in the coding regions. Among the six SNPs of YepMm, one at 21,330 bp located in the new ORF-29, which encoded phage capsid and scaffold; one at 37,921 bp of ORF-43 encoded hypothetical protein; and the rest four SNPs located at the direct repeats (DR) terminal regions (216, 217, 38,610, and 38,611 bp). The SNP at 21,330 bp located in the upstream activating sequence of tail tubular protein A (TTPA) in genome of phage Yep-phi; however, a new ORF-encoded phage capsid and scaffold generated by this SNP in the genome of YepMm (**Figure 3B**). The missense mutation of 21,330 bp caused the termination codon to change to Glu amino acid; 37,921 bp caused the Ile to change to Leu amino acid (**Figure 3C**).

Compared with the lytic phages for *Y. pestis* characterized previously, the nine available genome sequences could be divided into two subgroups (**Figure 2**). The genome of YepMm clustered with the bacteriophages Yep-phi, Berlin (GenBank accession number, AM183667.1), YpP-G (JQ965702.1), and Yepe2 (EU734170.1), and these bacteriophages comprised subgroup A. The other subgroup comprised *Yersinia* phage YpP-R (GenBank accession number, JQ965701.1), *Yersinia* phage_Y (JQ957925.1), *Yersinia* phage phiA1122 (AY247822.1), *Yersinia* phage_R (JX000007.1), *Yersinia* phage YpP-Y (Q965700.1), and *Yersinia* phage YpsP-G (JQ965703.1).

### Lytic Abilities and Efficiency of the Bacteriophages YepMm and Yep-phi on the Host Strain EV76

Every half hour, the optical density at 600 nm (OD_600_) value was plotted to generate a growth curve for each group. The OD_600_ of EV76 increased initially and then decreased rapidly upon bacteriophage addition. The growth curve decreased more rapidly after infection with the bacteriophage YepMm compared with that in infection with the bacteriophage Yep-phi. With the initial concentration of MCF = 3.3, the OD_600_ of culture solution infected with bacteriophage YepMm began to descend at 2 h later compared with 4.3 h after being infected with bacteriophage Yep-phi. When with the initial concentration of MCF=1.0, the OD_600_ of culture solution infected with bacteriophage YepMm began to descend at 1.3 h later compared with 2.3 h after being infected with bacteriophage Yep-phi. Hence, the lytic ability of the bacteriophage YepMm was more efficient than that of the bacteriophage Yep-phi. Statistical analysis showed the difference is significant at the 0.05 level (**Table S3** and **Figures 1F, G**). The culture solution infected with bacteriophage YepMm lyse absolutely within three and half hours with the initial concentration of MCF=1.0, shorter than initial concentration of MCF=3.3 (almost within 10 h) (**Figures 1F, G**).

## Discussion

*Y. pestis* is the causative agent of plague. It emerged from the enteropathogen O:1b *Y. pseudotuberculosis* 3,000 years ago by losing many genes and the horizontal acquisition of several genetic elements (Wren, 2003). Lytic bacteriophages have been used as therapeutic and prophylactic agents for controlling bacterial infections. Over the past 100 years, lytic bacteriophages have been used for the diagnosis of *Y. pestis* infections and to identify plagues caused by *Y. pestis* (D’Herelle and Malone, 1927; Duckworth, 1976).

We isolated, for the first time, the lytic bacteriophages of *Y. pestis* from an epidemic-focus area of *Y. pestis* in China. Our study on the bacteriophage YepMm showed a very broad range of hosts for bacteria of the genus *Yersinia*. This range included all of the three human pathogenic *Yersinia* species: *Y. pestis*, *Y. pseudotuberculosis* (O:1a, O:1b, and O:14), and the highly pathogenic *Y. enterocolitica* bioserotype 1B/O:8. Even though the genomes of YepMm and Yep-phi are almost identical, they varied in their ability to lyse bacteria of the genus *Yersinia*. Analyses of the host range showed that YepMm could infect not only *Y. pestis* strains but also the strains of the highly pathogenic *Y. enterocolitica* bioserotype 1B/O:8 and several strains of *Y. pseudotuberculosis*. However, Yep-phi is a *Y. pestis*-specific lytic bacteriophage (Zhao et al., 2011). The different phage receptors for adsorption are one of the important reasons to different bacteriolytic efficacy (Liang et al., 2016; Zhao and Skurnik, 2016). Our findings suggest that a sense mutation of an upstream activating sequence of TTPA generate a new ORF, which may modify phage tail protein and cause differences in host sensitivity. TTPA has been described as a structural protein of a bacteriophage tail. It forms an attachment for tail spikes to mediate infection through sensing the deflection of side fibers upon cell-wall binding. During infection by bacteria, TPPA can bind with bacterial receptors to mediate bacteriophage adsorption and subsequent bacterial lysis (Hu et al., 2020; Pyra et al., 2020a; Pyra et al., 2020b). If differences occur specifically in the genes encoding the tail fibers, then recognition of the cell target will change (Vacheron et al., 2021). How a mutation in the upstream activating sequence of TTPA modifies its expression merits investigation.

We discovered that YepMm could form plaques on two more strains (*Y. enterocolitica* YE92010 and *Y. pseudotuberculosis* 53512) at 37°C than at 25°C (**Table 1**). This finding was likely because of the receptors being recognized specifically at a higher temperature, with a reduced ability of the bacteriophage (and parental bacteriophage) to infect and grow on host strains at a lower temperature. Despite the almost identical genome sequences of the bacteriophages YepMm and Yep-phi, they varied in their ability to lyse host bacteria among *Yersinia* species, which suggests that they might use different receptors for adsorption. The bacteriophages Yep-phi and ϕA1122 have been used as a diagnostic agent *Y. pestis* infection (Hu et al., 2020). Unlike the bacteriophage YepMm, the bacteriophage Yep-phi infects *Y. pestis* exclusively and is inactive toward other *Yersinia* species, irrespective of the growth temperature (Zhao et al., 2011; Zhao and Skurnik, 2016); the phage A1122 only grows on *Y. pseudotuberculosis* at 37°C and not at 25°C. Obviously, the phage YepMm has the broadest host range. Interestingly, strains of the highly pathogenic *Y. enterocolitica* bioserotype 1B/O:8 differed markedly in their susceptibility to the bacteriophage YepMm and had a temperature-dependent response.

Bacteriophage control is the most environmentally friendly method used to eradicate pathogens from food products. The lytic properties and activity of the bacteriophage YepMm in controlling infection from *Yersinia* species will be studied in the future.

## Data Availability Statement

The datasets presented in this study can be found in online repositories. The names of the repository/repositories and accession number(s) can be found below: https://www.ncbi.nlm.nih.gov/genbank/, MW767996.

## Ethics Statement

The animal study was reviewed and approved by Ethics Committee of National Institute for Communicable Disease Control and Prevention, Chinese Center for Disease Control and Prevention.

## Author Contributions

JL, XW, SQ, RD preparing manuscript, writing, and correction this manuscript, JL, ZH, and XuL did designed figures. HJ, HZ, WW, DT, GF, XML, DL generated experimental data and wrote the manuscript. HM, MX, JY, JL, SQ, RD, HJ, XW conceived the work and critically review the manuscript. All authors contributed to the article and approved the submitted version.

## Funding

This work was supported by National Science and Technology Major Project (2018ZX10713-003-002, 2018ZX10713-001-002).

## Conflict of Interest

The authors declare that the research was conducted in the absence of any commercial or financial relationships that could be construed as a potential conflict of interest.

## Supplementary Material

Supplementary Figure 1 One-step growth curve of YepMm with MOI =10. Each data is shown as mean ± SD from three biological experiments.

Supplementary Table 1 Titers of the phage YepMm under different MOI.

Supplementary Table 2 Structural comparison of the ORFs of bacteriophages Yep-phi and YepMm.

Supplementary Table 3 Statistical analysis between YepMm and Yep-phi at two MCFs with different time points.

## References

[B1] Bergh O. Borsheim K. Y. Bratbak G. Heldal M. (1989). High Abundance of Viruses Found in Aquatic Environments. Nature 340, 467–468. 10.1038/340467a0 2755508

[B2] Berthiaume L. Ackermann H. W. (1977). Classification of Actinophages. Pathol. Biol. (Paris) 25, 195–201. 323790

[B3] Chhibber S. Kaur T. Sandeep K. (2013). Co-Therapy Using Lytic Bacteriophage and Linezolid: Effective Treatment in Eliminating Methicillin Resistant Staphylococcus Aureus (MRSA) From Diabetic Foot Infections. PloS One 8, e56022. 10.1371/journal.pone.0056022 23418497 PMC3572146

[B4] D’Herelle F. Malone R. H. (1927). A Preliminary Report of Work Carried Out by the Cholera Bacteriophage Enquiry. Ind. Med. Gaz 62, 614–616. 29010807 PMC5197969

[B5] Doub J. B. (2020). Bacteriophage Therapy for Clinical Biofilm Infections: Parameters That Influence Treatment Protocols and Current Treatment Approaches. Antibiotics (Basel) 9, 799. 10.3390/antibiotics9110799 PMC7697957 33198058

[B6] Duckworth D. H. (1976). “Who Discovered Bacteriophage? Bacteriol Rev. 40, 793–802. 10.1128/br.40.4.793-802.1976 795414 PMC413985

[B7] Flayhan A. Vellieux F. M. Lurz R. Maury O. Contreras-Martel C. Girard E.. (2014). Crystal Structure of Pb9, the Distal Tail Protein of Bacteriophage T5: A Conserved Structural Motif Among All Siphophages. J. Virol. 88, 820–828. 10.1128/JVI.02135-13 24155371 PMC3911636

[B8] Garcia E. Elliott J. M. Ramanculov E. Chain P. S. Chu M. C. Molineux I. J. (2003). The Genome Sequence of Yersinia Pestis Bacteriophage Phia1122 Reveals an Intimate History With the Coliphage T3 and T7 Genomes. J. Bacteriol 185, 5248–5262. 10.1128/JB.185.17.5248-5262.2003 12923098 PMC181008

[B9] Ge P. Xi J. Ding J. Jin F. Zhang H. Guo L.. (2015). Primary Case of Human Pneumonic Plague Occurring in a Himalayan Marmot Natural Focus Area Gansu Province, China. Int. J. Infect. Dis. 33, 67–70. 10.1016/j.ijid.2014.12.044 25555623

[B10] Gorski A. Miedzybrodzki R. Borysowski J. Weber-Dabrowska B. Lobocka M. Fortuna W.. (2009). Bacteriophage Therapy for the Treatment of Infections. Curr. Opin. Investig. Drugs 10, 766–774. 10.1117/12.895292 19649921

[B11] Hardy J. M. Dunstan R. A. Grinter R. Belousoff M. J. Wang J. Pickard D.. (2020). The Architecture and Stabilisation of Flagellotropic Tailed Bacteriophages. Nat. Commun. 11, 3748. 10.1038/s41467-020-17505-w 32719311 PMC7385642

[B12] Hu M. Zhang H. Gu D. Ma Y. Zhou X. (2020). Identification of a Novel Bacterial Receptor That Binds Tail Tubular Proteins and Mediates Phage Infection of Vibrio Parahaemolyticus. Emerg. Microbes Infect. 9, 855–867. 10.1080/22221751.2020.1754134 32306848 PMC7241545

[B13] Kiljunen S. Datta N. Dentovskaya S. V. Anisimov A. P. Knirel Y. A. Bengoechea J. A.. (2011). Identification of the Lipopolysaccharide Core of Yersinia Pestis and Yersinia Pseudotuberculosis as the Receptor for Bacteriophage Phia1122. J. Bacteriol 193, 4963–4972. 10.1128/JB.00339-11 21764935 PMC3165662

[B14] Kiljunen S. Vilen H. Savilahti H. Skurnik M. (2003). Transposon Mutagenesis of the Phage Phi YeO3-12. Adv. Exp. Med. Biol. 529, 245–248. 10.1007/0-306-48416-1_47 12756765

[B15] Liang J. Li X. Zha T. Chen Y. Hao H. Liu C.. (2016). DTDP-Rhamnosyl Transferase RfbF, Is a Newfound Receptor-Related Regulatory Protein for Phage phiYe-F10 Specific for Yersinia Enterocolitica Serotype O:3. Sci. Rep. 6, 22905. 10.1038/srep22905 26965493 PMC4786787

[B16] Moojen D. J. F. (2013). Exploring New Strategies for Infection Treatment. J. Bone Joint Surg. Am. 95 (2), e11. 10.2106/JBJS.L.01419 23324967

[B17] Mukerjee S. Roy U. K. Rudra B. C. (1963). Studies on Typing of Cholera Vibrios by Bacteriophage. V. Geographical Distribution of Phage-Types of Vibrio Cholerae. Ann. Biochem. Exp. Med. 23, 523–530. 14154194

[B18] Muniesa M. Lucena F. Blanch A. R. Payan A. Jofre J. (2012). Use of Abundance Ratios of Somatic Coliphages and Bacteriophages of Bacteroides Thetaiotaomicron GA17 for Microbial Source Identification. Water Res. 46, 6410–6418. 10.1016/j.watres.2012.09.015 23039916

[B19] Pyra A. Filik K. Szermer-Olearnik B. Czarny A. Brzozowska E. (2020a). New Insights on the Feature and Function of Tail Tubular Protein B and Tail Fiber Protein of the Lytic Bacteriophage Phiyeo3-12 Specific for Yersinia Enterocolitica Serotype O:3. Molecules 25, 4392. 10.3390/molecules25194392 PMC7582827 32987777

[B20] Pyra A. Urbanska N. Filik K. Tyrlik K. Brzozowska E. (2020b). Biochemical Features of the Novel Tail Tubular Protein A of Yersinia Phage Phiyeo3-12. Sci. Rep. 10, 4196. 10.1038/s41598-020-61145-5 32144374 PMC7060351

[B21] Rashid M. H. Revazishvili T. Dean T. Butani A. Verratti K. Bishop-Lilly K. A.. (2012). A Yersinia pestis-specific, Lytic Phage Preparation Significantly Reduces Viable Y. pestis on various hard surfaces experimentally contaminated with the bacterium. Bacteriophage 2, 168–177. 10.4161/bact.22240 23275868 PMC3530526

[B22] Shubeita H. E. Sambrook J. F. McCormick A. M. (1987). Molecular Cloning and Analysis of Functional cDNA and Genomic Clones Encoding Bovine Cellular Retinoic Acid-Binding Protein. Proc. Natl. Acad. Sci. U.S.A. 84, 5645–5649. 10.1073/pnas.84.16.5645 3039499 PMC298919

[B23] Vacheron J. Heiman C. M. Keel C. (2021). Live Cell Dynamics of Production, Explosive Release and Killing Activity of Phage Tail-Like Weapons for Pseudomonas Kin Exclusion. Commun. Biol. 4, 87. 10.1038/s42003-020-01581-1 33469108 PMC7815802

[B24] Wang H. Cui Y. Wang Z. Wang X. Guo Z. Yan Y.. (2011). A Dog-Associated Primary Pneumonic Plague in Qinghai Province, China. Clin. Infect. Dis. 52, 185–190. 10.1093/cid/ciq107 21288842

[B25] Wang X. Wei X. Song Z. Wang M. Xi J. Liang J.. (2017). Mechanism Study on a Plague Outbreak Driven by the Construction of a Large Reservoir in Southwest China (Surveillance From 2000-2015). PloS Negl. Trop. Dis. 11, e0005425. 10.1371/journal.pntd.0005425 28257423 PMC5352140

[B26] Wren B. W. (2003). The Yersiniae–a Model Genus to Study the Rapid Evolution of Bacterial Pathogens. Nat. Rev. Microbiol. 1, 55–64. 10.1038/nrmicro730 15040180

[B27] Zhang Z. Tian C. Zhao J. Chen X. Wei X. Li H.. (2018). Characterization of Tail Sheath Protein of N4-Like Phage Phiaxp-3. Front. Microbiol. 9, 450. 10.3389/fmicb.2018.00450 29599760 PMC5862860

[B28] Zhao X. Skurnik M. (2016). Bacteriophages of Yersinia Pestis. Adv. Exp. Med. Biol. 918, 361–375. 10.1007/978-94-024-0890-4_13 27722870

[B29] Zhao X. Wu W. Qi Z. Cui Y. Yan Y. Guo Z.. (2011). The Complete Genome Sequence and Proteomics of Yersinia Pestis Phage Yep-Phi. J. Gen. Virol. 92, 216–221. 10.1099/vir.0.026328-0 20943893

