# Supplementary figures and images for: A Lytic Yersina pestis Bacteriophage Obtained From the Bone Marrow of Marmota himalayana in a Plague-Focus Area in China
Source: Front Cell Infect Microbiol. 2021 Jul 8;11:700322. doi: 10.3389/fcimb.2021.700322 (PMC8297710; doi:10.3389/fcimb.2021.700322)

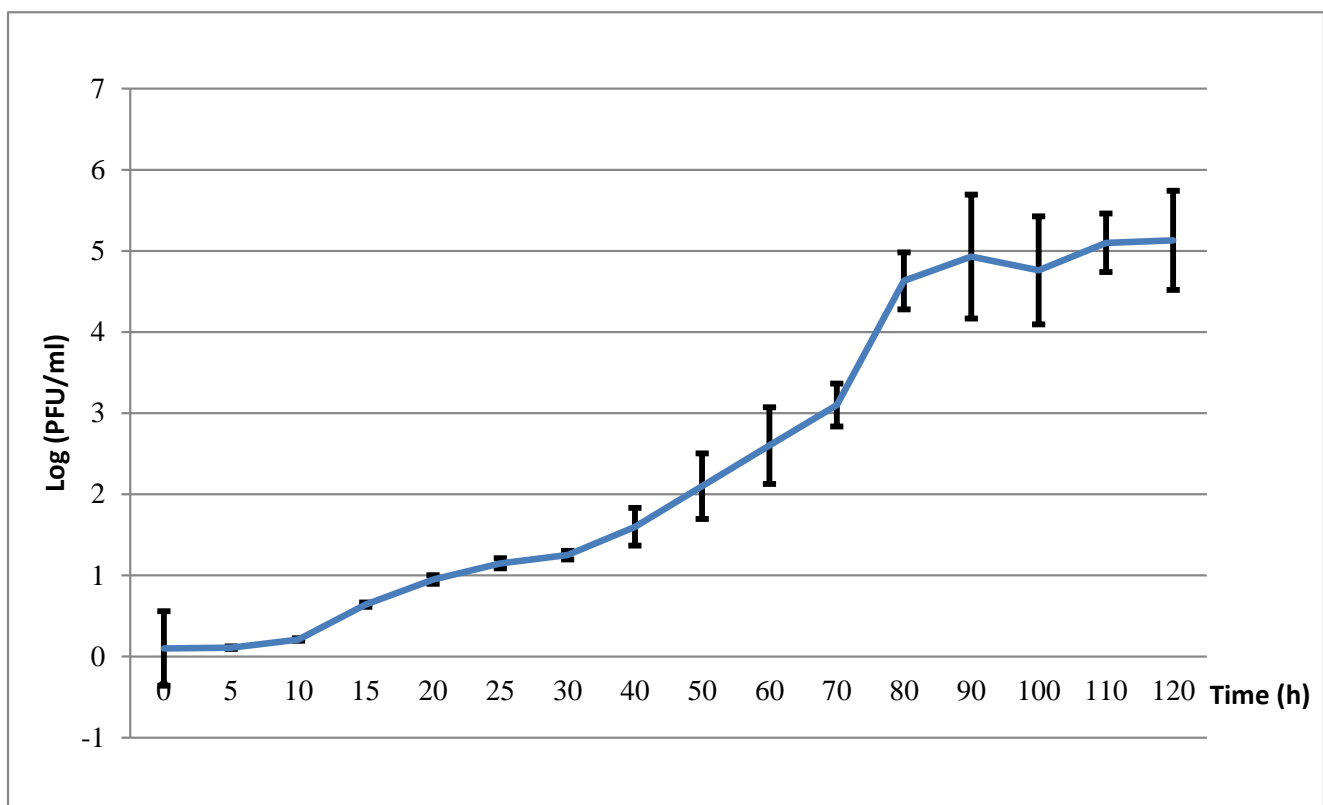

Supplement: Supplementary Figure 1 — One-step growth curve of YepMm with MOI =10. Each data is shown as mean ± SD from three biological experiments. [file Image_1.pdf]
