# Supplementary material for: A Lytic Yersina pestis Bacteriophage Obtained From the Bone Marrow of Marmota himalayana in a Plague-Focus Area in China
Source: Front Cell Infect Microbiol. 2021 Jul 8;11:700322. doi: 10.3389/fcimb.2021.700322 (PMC8297710; doi:10.3389/fcimb.2021.700322)
Supplement: Supplementary Table 1 — Titers of the phage YepMm under different MOI. [file Table_1.pdf]

Supplementary table 1: Titers of the phage under different MOIs

Group	MOI	Number of Bacterial (CFU/ml)	Number of phages (PFU/ml)	Phage titers after incubated for 3 h (PFU/ml)
1	100	10^7	10^9	4.4×10^9
2	10	10^8	10^9	$5.3\times10^{10*}$
3	1	10^8	10^8	3.5×10^{10}
4	0.1	10^8	10^7	1.1×10^{10}
5	0.01	10^8	10^6	6.2×10^9
6	0.001	10^8	10^5	1.4×10^9

* (phage/bacteria = 0.001, 0.01, 0.1, 1, 10 and 100)

At the MOI of 10, phage reached maximum titers.
