# Supplementary material for: A Lytic Yersina pestis Bacteriophage Obtained From the Bone Marrow of Marmota himalayana in a Plague-Focus Area in China
Source: Front Cell Infect Microbiol. 2021 Jul 8;11:700322. doi: 10.3389/fcimb.2021.700322 (PMC8297710; doi:10.3389/fcimb.2021.700322)
Supplement: Supplementary Table 2 — Structural comparison of the ORFs of bacteriophages Yep-phi and YepMm. [file Table_2.pdf]

Supplementary table 2: Structural comparison of the ORFs of bacteriophages Yep-phi and YepMm.

Yep-phi	YePMm
>HQ333270.1 ORF_1: 990:1220	>YePMm_ORF1: 990:1220
>HQ333270.1 ORF_2: 1291:2418	>YePMm_ORF2: 1291:2418
>HQ333270.1 ORF_3: 2492:5143	>YePMm_ORF3: 2492:5143
>HQ333270.1 ORF_4: 5257:5406	>YePMm_ORF4: 5257:5406
>HQ333270.1 ORF_5: 5406:5663	>YePMm_ORF5: 5406:5663
>HQ333270.1 ORF_6: 5794:6822	>YePMm_ORF6: 5794:6822
>HQ333270.1 ORF_7: 7008:7271	>YePMm_ORF7: 7008:7271
>HQ333270.1 ORF_8: 7271:7585	>YePMm_ORF8: 7271:7585
>HQ333270.1 ORF_9: 7673:7864	>YePMm_ORF9: 7673:7864
>HQ333270.1 ORF_10: 7932:8639	>YePMm_ORF10: 7932:8639
>HQ333270.1 ORF_11: 8659:9108	>YePMm_ORF11: 8659:9108
>HQ333270.1 ORF_12: 9108:9563	>YePMm_ORF12: 9108:9563
>HQ333270.1 ORF_13: 9732:11441	>YePMm_ORF13: 9732:11441
>HQ333270.1 ORF_14: 11516:11728	>YePMm_ORF14: 11516:11728
>HQ333270.1 ORF_15: 11742:12038	>YePMm_ORF15: 11742:12038
>HQ333270.1 ORF_16: 12133:13734	>YePMm_ORF16: 12133:13734
>HQ333270.1 ORF_17: 13807:14241	>YePMm_ORF17: 13807:14241
>HQ333270.1 ORF_18: 14598:14927	>YePMm_ORF18: 14598:14927
>HQ333270.1 ORF_19: 14947:15240	>YePMm_ORF19: 14947:15240
>HQ333270.1 ORF_20: 15240:15449	>YePMm_ORF20: 15240:15449
>HQ333270.1 ORF_21: 15446:15625	>YePMm_ORF21: 15446:15625
>HQ333270.1 ORF_22: 15622:16536	>YePMm_ORF22: 15622:16536
>HQ333270.1 ORF_23: 16670:16915	>YePMm_ORF23: 16670:16915
>HQ333270.1 ORF_24: 16933:17214	>YePMm_ORF24: 16933:17214
>HQ333270.1 ORF_25: 17229:17501	>YePMm_ORF25: 17229:17501
>HQ333270.1 ORF_26: 17516:19123	>YePMm_ORF26: 17516:19123
>HQ333270.1 ORF_27: 19197:20111	>YePMm_ORF27: 19197:20111
>HQ333270.1 ORF_28: 20242:21285	>YePMm_ORF28: 20242:21285
>HQ333270.1 ORF_29: 21493:22083	>YePMm_ORF29: 21255:21419
>HQ333270.1 ORF_30: 22110:24488	>YePMm_ORF30: 21493:22083
>HQ333270.1 ORF_31: 24572:24982	>YePMm_ORF31: 22110:24488
>HQ333270.1 ORF_32: 24986:25573	>YePMm_ORF32: 24572:24982
>HQ333270.1 ORF_33: 25609:27888	>YePMm_ORF33: 24986:25573
>HQ333270.1 ORF_34: 27912:31859	>YePMm_ORF34: 25609:27888
>HQ333270.1 ORF_35: 31942:33651	>YePMm_ORF35: 27912:31859
>HQ333270.1 ORF_36: 33704:33910	>YePMm_ORF36: 31942:33651
>HQ333270.1 ORF_37: 33903:34172	>YePMm_ORF37: 33704:33910
>HQ333270.1 ORF_38: 34271:34720	>YePMm_ORF38: 33903:34172
>HQ333270.1 ORF_39: 34713:35309	>YePMm_ORF39: 34271:34720
>HQ333270.1 ORF_40: 35325:37082	>YePMm_ORF40: 34713:35309

>HQ333270.1 ORF_41: 37113:37571	>YePMm_ORF41: 35325:37082
>HQ333270.1 ORF_42: 37947:38102	>YePMm_ORF42: 37113:37571
	>YePMm_ORF43: 37843:37998
