# Supplementary material for: A Lytic Yersina pestis Bacteriophage Obtained From the Bone Marrow of Marmota himalayana in a Plague-Focus Area in China
Source: Front Cell Infect Microbiol. 2021 Jul 8;11:700322. doi: 10.3389/fcimb.2021.700322 (PMC8297710; doi:10.3389/fcimb.2021.700322)
Supplement: Supplementary Table 3 — Statistical analysis between YepMm and Yep-phi at two MCFs with different time points. [file Table_3.pdf]

Supplementary table 3: Statistical analysis between YepMm and Yep-phi at two MCFs with different time points

MCF	Time (h)	F (Sig.) Between Groups	(I) group	(J) group	Sig.	95% Confidence Interval Lower Bound	95% Confidence Interval Upper Bound
1.0	1.3	123.75 (0.00)*	1	2	0.02*	0.004	0.03
				3	0.00*	0.07	0.09
			2	1	0.02*	-0.03	0.00
				3	0.00*	0.05	0.08
			3	2	0.00*	-0.08	-0.05
				1	0.00*	-0.09	-0.07
	2.3	326.54 (0.00)*	1	2	0.00*	0.12	0.16
				3	0.00*	0.20	0.24
			2	1	0.00*	-0.16	-0.12
				3	0.00*	0.05	0.10
			3	2	0.00*	-0.10	-0.05
				1	0.00*	-0.24	-0.20
3.3	2	25.17 (0.01)*	1	2	0.47	-0.03	0.05
				3	0.00*	0.07	0.15
			2	1	0.47	-0.05	0.03
				3	0.00*	0.05	0.14
			3	1	0.00*	-0.15	-0.07
				2	0.00*	-0.14	-0.05
	4.3	74.74 (0.00)*	1	2	0.01*	0.03	0.15
				3	0.00*	0.23	0.35
			2	1	0.01*	-0.15	-0.03
				3	0.00*	0.14	0.27
			3	1	0.00*	-0.35	-0.23
				2	0.00*	-0.27	-0.14

*. The mean difference is significant at the 0.05 level.

Group 1: culture solution without phages infected

Group 2: culture solution infected with bacteriophage Yep-phi

Group 3: culture solution infected with bacteriophage YepMm
